# Design of Functionalized Photon Sieves for the Detection of Biomarkers in Running Fluids

**DOI:** 10.3390/s26020409

**Published:** 2026-01-08

**Authors:** Veronica Pastor-Villarrubia, Luis Pablo Canul-Solis, Luis Carlos Ortiz-Dosal, José Gabriel Roberto Hernández-Arteaga, Eleazar Samuel Kolosovas-Machuca, Luis Miguel Sanchez-Brea, Javier Alda

**Affiliations:** 1 Applied Optics Complutense Group, Optics Department, Faculty of Optics and Optometry, Universidad Complutense de Madrid, C/Arcos de Jalón, 118, 28037 Madrid, Spain; veronica.pastor@ucm.es (V.P.-V.); samuel.kolosovas@uaslp.mx (E.S.K.-M.); 2 Facultad de Ciencias, Universidad Autónoma de San Luis Potosí, 1570 Parque Chapultepec Ave, San Luis Potosí 78295, Mexico; luispablo.canulsolis@student.kuleuven.be; 3 Faculty of Science Engineering, Katholieke Universiteit Leuven, Kasteelpark Arenberg 1 bus 2200, 3001 Leuven, Belgium; 4 Unidad Académica de Ingeniería I, Universidad Autónoma de Zacatecas, 801 López Velarde St, Zacatecas 98000, Mexico; ortiz.dosal.lc@uaz.edu.mx; 5 Coordinación para la Innovación y Aplicación de la Ciencia y la Tecnología, Universidad Autónoma de San Luis Potosí, 550 Sierra Leona Ave, San Luis Potosí 78210, Mexico; roberto.hernandez@uaslp.mx; 6 Applied Optics Complutense Group, Optics Department, Faculty of Physics, Universidad Complutense de Madrid, Plaza de las Ciencias, 1, 28040 Madrid, Spain; optbrea@ucm.es

**Keywords:** spectral sensing, photon sieve, diffractive optical elements, permeable optics, optical detection, aluminum substrate, functionalization

## Abstract

In this work, we present the design of a prototype fluid analyzer based on photon sieves, permeable diffractive optical elements capable of focusing light through diffraction. The photon sieve comprises a spatial distribution of circular apertures patterned onto an aluminum substrate, which provides intrinsic fluid permeability and functions as either a lens or a mirror. In our approach, the aluminum surface is chemically functionalized to detect a specific biomolecular marker—human serum albumin—whose interaction with the surface induces measurable changes in the spectral reflectance. The operating wavelength is selected to maximize the reflectance contrast produced by the presence of the biomarker. The optical set-up is configured such that the light source and detector lie in the same plane when the photon sieve operates in reflection. A combined geometrical and diffractive analysis is conducted to optimize their positions. Upon detection of the biomarker, the measured signal decreases to 0.43 of its initial value prior to biomarker binding. These results highlight photon sieves as a promising platform for the development of compact, lightweight, and low-cost optical chemical sensors for running fluids.

## 1. Introduction

The ability to analyze fluids is essential in various fields, including biomedical [1], environmental [2,3,4], and industrial applications [5]. In the biomedical sector, there is a growing interest in non-invasive methods that clear the way for early detection and diagnosis of diseases such as diabetes, renal disorders, cardiovascular conditions, and metabolic syndromes [6]. In industrial environments, this monitoring can lead to significant improvements in the early detection of faults and in the implementation of corrective actions for environmental control [7]. However, fluid analysis often requires expensive instrumentation. Even when more affordable equipment is available, data acquisition and interpretation usually demand trained personnel, which increases both cost and complexity while limiting the accessibility of such tools.

Over recent decades, the application of optical techniques for the development of sensors across domains ranging from biomedical to industrial has gained great relevance. Examples include sensors based on optical fibers [8,9,10,11,12], waveguides [13,14,15,16], resonant structures [17,18,19], surface plasmon resonances [20,21,22], photonic crystals [23,24], and photovoltaic structures [25]. These devices can detect variations in optical parameters—such as refractive index, intensity, reflectance, absorbance, color, or lifetime—associated with concentration changes or the presence of one or several target substances. Such sensors have been implemented for environmental monitoring, clinical diagnosis, and industrial process control [26].

Within the biomedical context, particularly interesting developments include biosensors based on microneedles, functional tattoos, and skin-integrated devices [27,28,29]. These systems are non-invasive or minimally invasive, rapid, and highly portable. They enable continuous monitoring of physiological parameters such as glucose through the interstitial fluid [30]. The main drawback of these novel sensors lies in their complex architectures and the use of specialized materials, which currently make them expensive and difficult to scale for commercial production. Moreover, some of the previously described techniques are refractometric in nature: detection relies on the accurate measurement of the index of refraction. This strategy makes the sensitivity of these systems, expressed in refractive index units, strongly affected by environmental changes in temperature and pressure [22,25,31,32]. For this reason, it remains of great interest to explore alternative optical strategies capable of monitoring variations in optical parameters at lower cost and through easily manufacturable devices. Diffractive optical elements (DOEs)—in particular photon sieves (PSs)—represent an attractive alternative due to their compactness, high optical resolution, and permeability.

In previous works, the design of permeable diffractive lenses based on PSs was validated—through both simulation [33], using a dedicated Python (v. 3.12.4) package [34], and implementation in a spatial light modulator (SLM) [35]. In the present study, we propose and evaluate an optical biosensor that, by analyzing the reflectometric response of a functionalized PS operating in reflection, can detect the presence of a chemical marker of interest. The sensing element is functionalized through a proven chemical process that has been realized on high-purity aluminum substrates [36]. Other authors have used similar procedures for the functionalization of metal oxides and some more-biocompatible metals [37,38,39], meaning that, when mirror reflectance is preserved, the optical set-up presented here can be applied to a variety of materials and biomarkers.

This paper is organized as follows: After this Introduction Section, we have included a Materials and Methods Section (Section 2) that has been split in three subsections specially devoted to the main elements of the proposed device. First, in Section 2.1, we briefly specify how the functionalization is performed. We also present the spectral reflectance obtained from this process. This analysis is conducted to find an optimum wavelength of operation. This functionalized surface is integrated within a permeable diffractive element—a photon sieve—that is optimized for reflection as explained in Section 2.2. The whole optical set-up is analyzed in Section 2.3 to justify how the emitter and detector are arranged on the same plane, and how the system is optically adapted for biosensing purposes. After selecting some geometrical parameters of the detector and source, the optical signals delivered by the device are analyzed in Section 3 to enable the detection of the molecules of interest. Finally, the main conclusions of this paper are presented in Section 4 to demonstrate how this system can be considered as a proof-of-concept optical sensor for the analysis of running fluids.

## 2. Materials and Methods

The optical set-up of this design is organized around the functionalized photon sieve mask. From an optical point of view, we look for the spectral location where the reflectance of the mask varies the most when the biomarker is bound. Therefore, the analysis of the spectral response of the functionalized surface justifies the selection of the optical frequency, ν, used in the sensor design, where $\nu =c/\lambda_{0}$, with *c* being the speed of light in vacuum and $\lambda_{0}$ the wavelength in vacuum. As far as diffraction is based on optical path differences, the calculation of the optical performance of the photon sieve must be made considering the wavelength in the medium under analysis. Then, we present here how to design an optimum PS working in reflection. The additional optical elements used to compact the device are also described in this section. These elements should accommodate the emitter and detector on the same plane to ease the fabrication of the system, taking advantage of the antiprincipal plane concept of geometrical optics. We choose a laser diode operating at the wavelength obtained from the reflectance analysis. Light enters the pipe where the liquid of interest runs through a flat window and illuminates the PS under almost normal incidence. The reflected light is collected by the detector located on the same plane as the emitter. Meanwhile, the transmitted light is received by another detector that generates a reference signal.

### 2.1. Surface Functionalization and Spectroscopic Characterization

**Figure 1:**
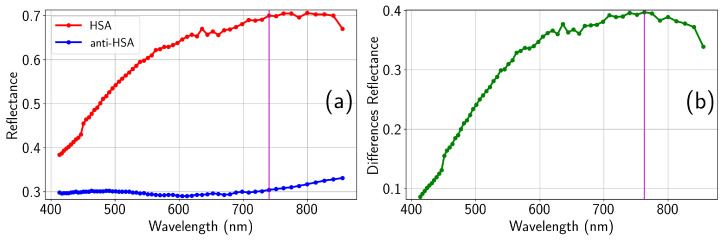
(**a**) Spectral reflectance obtained for HSA (closed red circles) and Anti-HSA (closed blue circles). (**b**) Differences in reflectance of the spectra shown in (**a**) (closed green circles). The vertical line at λ=763 nm identifies the wavelength where the maximum difference in reflectance occurs. The reflectances at this wavelength are $\rho_{\mathrm{HSA}}=0.705$ and $\rho_{\mathrm{Anti}-\mathrm{HSA}}=0.308$.

The functionalization process modifies the surface by attaching adequate organic functional groups required for the bonding and immobilization of biomolecules. In this work, high-purity aluminum substrates (99.999%) with an area of approximately 1 ${\mathrm{cm}}^{2}$ were functionalized through a five-step process [36]. (i) The substrate cleaning was performed using deionized water, isopropyl alcohol (protic solvent), and acetone (aprotic solvent) in the sequence water → isopropyl alcohol → water → acetone → water, followed by nitrogen drying. (ii) The surface oxidation stage was carried out by immersing the samples in a 5% aqueous KOH solution for 30 min, having proven more effective than acidic alternatives. (iii) In the silanization step, 3-aminopropyltriethoxysilane (APTES) molecules interact with the hydroxyl groups bound to the aluminum surface in the previous step. This is achieved by immersing the samples in a 10% *v*/*v* ethanolic solution of APTES for 4 h at room temperature, followed by rinsing with ethanol and drying with nitrogen. (iv) To reinforce the silane layer, crosslinking was performed by immersing the samples in a 1.25% *v*/*v* glutaraldehyde solution in phosphate-buffered saline (PBS), followed by rinsing with PBS. And (v) to attach the target protein, the functionalized surface was exposed to a 50% *v*/*v* human serum albumin (HSA)/PBS solution for 24 h. To evaluate the system’s ability to detect specific antigen–antibody interactions, a subset of the functionalized samples was subsequently incubated in a 50% *v*/*v* anti-HSA/PBS solution for an additional 24 h. Spectroscopy and spectroscopic ellipsometric techniques were then employed to evaluate changes in the optical response, validating the effectiveness of the functionalization process [36]. Especially important are the results obtained for the spectral reflectance before (HSA) and after the addition of anti-human serum albumin (Anti-HSA) to the functionalized substrates (see Figure 1).

Within the visible and near-infrared range, we can see how the maximum difference in reflectance happens around $\lambda_{0}=763\;\mathrm{nm}$, suggesting the optimum wavelength at which the sensor should exhibit a better performance to distinguish between HSA and anti-HSA states. It is important to note that this wavelength refers to the optical response of the sample measured in air, which we will consider as the wavelength in vacuum. In fact, when representing reflectance vs. frequency, the spectral response does not change with the index of refraction of the supersubstrate, unless nonlinear conversion is at work. The situation is different when computing the optical path lengths of the light trajectories. In this case, the actual wavelength within the medium should be considered, knowing that

(1) $$\lambda =\lambda_{0}/n,$$

with $\lambda_{0}$ being the wavelength in vacuum, and *n* the index of refraction of the media in which light propagates. In our case, this discussion is critical, because it affects the focal length of the PS used as a permeable diffractive optical element. As previously determined, we choose a light source emitting at $\lambda_{0}=763$ nm. However, as the design of the PS relies on the adjustment of optical paths, we have to use the actual wavelength within the media, which is shorter than in vacuum. In a realistic scenario, such as immersion in human serum—with a refractive index in the range of 1.33–1.36—assuming a refractive index of n=1.35 and using Equation (1), the wavelength in the medium is approximately λ=565 nm.

### 2.2. Design of the Photon Sieve

The central optical component of our device is a permeable diffractive optical element known as a photon sieve. This element is an optimized variation of a Fresnel zone lens. The optimization process has been previously analyzed and characterized in the optical bench, showing good agreement between the expected results obtained from simulations and the experimental values when the PS is realized using a spatial light modulator [33,35].

To better understand the involved geometrical parameters, we may recall the Fresnel zone principles that are at the base of the proposed design. In a Fresnel Zone Plate (FZP), the last Fresnel zone, labeled as *N*, has a radius given by the following equation:

(2) $$r_{N}=\sqrt{N\lambda f^{\prime }},$$

where λ is the wavelength of operation and $f^{\prime }$ is the focal length of the FZP. Following the reasoning of Section 2.1, λ=565 nm. Assuming that the total diameter of the PS is $D_{\mathrm{PS}}=2r_{N}$, we can obtain the total number of Fresnel zones *N* as

(3) $$N=\frac{D_{\mathrm{PS}}^{2}}{4\lambda f^{\prime }}.$$

Additionally, the minimum manufacturable radius $R_{\min}$ imposes a constraint on the smallest zone width. This means that the circular hole should occupy the band assigned to the last Fresnel zone. The width of this outermost Fresnel zone is

(4) $$\Delta r=r_{N+1}-r_{N}=\left(\sqrt{N+1}-\sqrt{N}\right)\sqrt{\lambda f^{\prime }}.$$

Using a first-order Taylor expansion for large *N*, this simplifies to

(5) $$\Delta r\approx \frac{1}{2}\sqrt{\frac{\lambda f^{\prime }}{N}}.$$

Within this approximation and by setting $\Delta_{r}=R_{\min}/2$, we obtain the maximum number of Fresnel zones as

(6) $$N=\frac{\lambda f^{\prime }}{R_{\min}^{2}}.$$

Combining Equations (3) and (6), we can relate the focal length of the PS, $f_{\mathrm{PS}}^{\prime }$, to its geometric and manufacturable parameters as follows:

(7) $$f_{\mathrm{PS}}^{\prime }=\frac{D_{\mathrm{PS}}R_{\min}}{2\lambda }.$$

A practical realization of this element has a diameter of $D_{\mathrm{PS}}=1$ cm, and a minimum hole radius of $R_{\min}=30$ μm, which produces a focal length of the PS of approximately $f_{\mathrm{PS}}^{\prime }=266$ mm. Then, F/#, defined as the ratio between diameter and focal length, is *f*:26.6. Such a large value assures that the device operates under almost normal incidence, meaning that the geometric shifts due to the closing window of the tube and polarization selectivity can be neglected.

**Figure 2:**
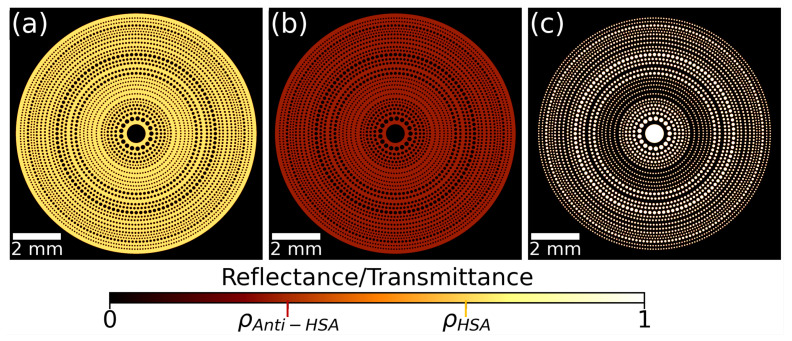
Reflectance and transmittance masks of the photon sieve optimized for maximizing the irradiance at the focal region when working in reflection mode. (**a**,**b**) Reflectance masks of the photon sieve for the HSA and anti-HSA states, respectively. (**c**) Transmittance mask of the photon sieve. The numerical values, normalized, are $\rho_{\mathrm{HSA}}=0.705$ and $\rho_{\mathrm{Anti}-\mathrm{HSA}}=0.308$. The transmittance of the open apertures is τ=1.

Optimized PSs can be generated using different approaches where the focused irradiance, permeability, depth of focus, or other optical or geometrical parameters are considered and combined in appropriate merit functions [33]. In this contribution, we have used a Ring-by-Ring design. This format iteratively maximizes the irradiance in the focal region when adding one ring at a time made of equal-diameter circular holes. The algorithm starts with the central hole and add the rings in ascending order of radial position. This Ring-by-Ring method offers the best compromise between the optimization of the depth of focus and the encircled energy around the focal point where a detector should be placed [35]. Given that we want the optical signal to be as large as possible, in this analysis, we paid full attention to the radiometric optimization. Therefore, permeability was not considered in the process, resulting in a value of 23.85%, meaning that almost 1/4 of the fluid crosses the PS. The result of this optimization is presented in Figure 2, where we have represented the reflectance of the two detection states (HSA in Figure 2a, and anti-HSA in Figure 2b), and the transmission of the permeable mask (see Figure 2c). We have used the Python package “Diffractio” [34] to perform the calculations. So far, this package only works in transmission. Therefore, the reflective mask has been modeled by placing circular stops instead of circular apertures.

### 2.3. Optical Design of the Sensor

**Figure 3:**
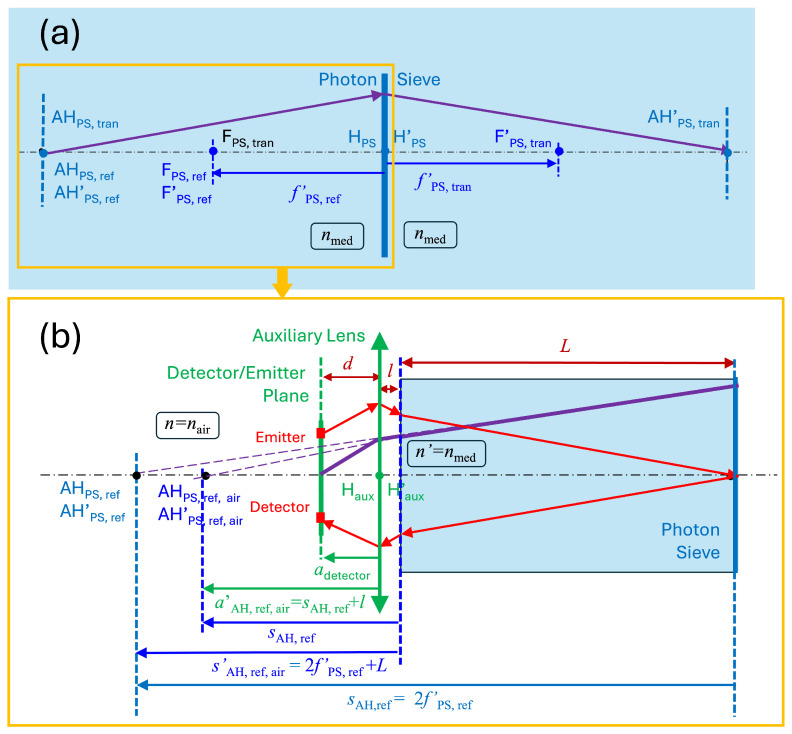
(**a**) Antiprincipal plane diagram for the PS when considering it to be immersed in the sensed media. AH_PS,ref_ and AH’_PS,ref_ are for the reflection mode, and AH_PS,tran_ and AH’_PS,tran_ are for the transmission mode. The focal distances of the PS are $f_{\mathrm{PS},\mathrm{ref}}^{\prime }$ and $f_{\mathrm{PS},\mathrm{tran}}^{\prime }$ for reflection and transmission, respectively. H_PS_ and H’_PS_ are the object and image principal planes, and F’_PS,ref_ and F’_PS,tran_ are the image focal points in reflection and transmission, respectively (F_PS,ref_ and F_PS,tran_ are the object focal points). (**b**) Antiprincipal plane diagram for the PS working in reflection, considering the effect of the plane interface between the air and medium, and the transformation produced by the auxiliary lens. The antiprincipal planes in the medium (AH_PS_, AH’_PS_) are shifted by the refraction at the plane interface, and finally moved with an auxiliary lens to the plane where the detector and emitter are located. This plot (**b**) corresponds to the portion working in reflection in plot (**a**), where all the relevant elements for a geometrical optics treatment are depicted.

The design of our sensing device must accommodate both reflection and transmission operation modes, which inherently doubles the overall size of the device [40]. However, this dual operation mode has an intrinsic advantage: the optical path of the transmission and reflection is balanced and, if the liquid is homogeneous, any variation in absorption and/or scattering is compensated and the ratio between the reference (in transmission) and detection channels (in reflection) should not be affected. Optically, in reflection mode, the PS is treated as a concave mirror, while in transmission mode, it behaves as a thin lens. Figure 3a shows both operating modes for a PS immersed in the media. As previously stated, this situation modifies the wavelength (see Equation (1)) that is used in the diffractional calculation of the PS.

To ease the optomechanical design, we place the emitter and detector on the same plane. This means that the optical system should work under a lateral magnification of $\beta^{\prime }=-1$. This case corresponds to the location of the object and image antiprincipal planes in an optical system, AH and AH’, respectively. From a simple geometrical optics analysis, this condition is satisfied if the object and image distances with respect to the PS are twice its focal length. If we consider the PS to be working in reflection, the focal length is negative because the equivalent mirror is concave. However, when working in transmission, the focal length becomes positive, meaning that $f_{\mathrm{PS},\mathrm{ref}}^{\prime }=-f_{\mathrm{PS},\mathrm{tran}}^{\prime }$, where subscripts _ref_ and _tran_ are for the reflection and transmission modes, respectively. At the same time, for a reflective PS, the object and image antiprincipal planes coincide on the same location; meanwhile, they appear on both sides of the PS when working in transmission. These differences are described as

(8) $$\begin{array}{ccc} s_{{\mathrm{AH}}^{\prime },\mathrm{ref}}^{\prime }=s_{\mathrm{AH},\mathrm{ref}} & = & 2f_{\mathrm{PS},\mathrm{ref}}^{\prime }, \end{array}$$

(9) $$\begin{array}{ccc} s_{{\mathrm{AH}}^{\prime },\mathrm{tran}}^{\prime }=-s_{\mathrm{AH},\mathrm{tran}} & = & 2f_{\mathrm{PS},\mathrm{tran}}^{\prime }. \end{array}$$

The analysis conducted in this section uses the DIN sign convention.

The value of the focal length of the PS has been calculated when immersed in the sensed medium. However, for the real system, light enters the pipe toward the PS through a window that closes the tube, as depicted in Figure 3b. When we neglect the thickness of this window, we may model this situation as an interface separating the medium and air. This means that the principal points move along the axis due to this added refraction. This effect can be calculated using the object–distance relation for a flat interface:

(10) $$\frac{n}{s}=\frac{n^{\prime }}{s^{\prime }}.$$

If we assume that the window closing the pipe is at a distance *L* from the PS, the result is that the actual location of the antiprincipal planes in reflection moves toward the PS and becomes

(11) $$s_{\mathrm{AH},\mathrm{ref},\mathrm{air}}=\frac{1}{n_{\mathrm{med}}}\left(s_{\mathrm{AH},\mathrm{ref}}+L\right),$$

where *L* is positive and $s_{\mathrm{AH},\mathrm{ref}}$ is negative because $f_{\mathrm{PS},\mathrm{ref}}^{\prime }$ is also negative and larger in magnitude than *L*. When substituting this in Equation (10) to obtain Equation (11), we have considered that $s_{\mathrm{AH},\mathrm{ref},\mathrm{air}}^{\prime }=s_{\mathrm{AH},\mathrm{ref}}+L$. In this analysis, we have neglected the thickness of the transparent window closing the pipe.

A fluid analyzer should keep the length of the total system as short as possible. To compact the design, we add an auxiliary lens to image the plane where the emitter and detector are placed (see Figure 3b) to the location of the antiprincipal planes in air. The value of the focal length of this auxiliary system depends on the actual optomechanical constraints of the device. We may assume a separation, *l*, between the lens (considered as a thin lens) and the closing window of the tube, and also a separation between the source/detector plane and the lens, *d*, where *l* is positive and *d* is negative because the source and emitter are located to the left of the auxiliary lens. This means that the lens has to produce an image located at $a_{\mathrm{AH},\mathrm{ref},\mathrm{air}}^{\prime }=s_{\mathrm{AH},\mathrm{ref},\mathrm{air}}+l$, for an object placed at $a_{\mathrm{detector}}=d$. Taking into account the object–image relation for a thin lens, we obtain the following relation between the involved parameters:

(12) $$f_{\mathrm{aux}}^{\prime }=\frac{a_{\mathrm{detector}}\times \left[\frac{1}{n_{\mathrm{med}}}\left(2f_{\mathrm{PS},\mathrm{ref}}^{\prime }+L\right)+l\right]}{-\left[\frac{1}{n_{\mathrm{med}}}\left(2f_{\mathrm{PS},\mathrm{ref}}^{\prime }+L\right)+l\right]+a_{\mathrm{detector}}}.$$

For the case treated in this contribution, we have considered L=300 mm, l=20 mm, and d=−50 mm. After substitution, the variables given in Equations (11) and (12) and represented in Figure 3 are $f_{\mathrm{PS}}^{\prime }\;=\;-266$ mm, $s_{\mathrm{AH},\mathrm{ref}}\;=\;-532$ mm, $s_{\mathrm{AH},\mathrm{ref},\mathrm{air}}^{\prime }\;=\;-232$ mm, $s_{\mathrm{AH},\mathrm{ref},\mathrm{air}}\;=\;-171.9$ mm, $a_{\mathrm{detector}}\;=\;-50$ mm, and $a_{\mathrm{AH},\mathrm{ref},\mathrm{air}}^{\prime }\;=\;-151.9$ mm, and the focal length of the auxiliary lens is $f_{\mathrm{aux}}^{\prime }\;=\;74.5$ mm.

## 3. Analysis and Results

Previous contributions proposed alternative optimization strategies for PSs that explicitly balance optical and geometric objectives [33,40]. The main geometric parameters of interest are the transversal diameter of the PS, which we fixed as 10 mm, and the device’s permeability, which is quantified as the open-area fraction (perforated area) of the aluminum substrate. In this case, permeability has not been considered for the optimization, but it still provides quite a large value of 23.85%. The optical objectives are related to the strength of the optical signal, the detector-integrated irradiance at the observation plane, and the depth of focus (DOF), which is important to reduce defocus errors. The PS design presented here achieves a balanced compromise between these optical and geometric criteria.

A novelty in the design of the present PS is that the optimization is carried out for a reflective device, unlike previous works [33,35]. This change is a consequence of the functionalization of only one face of the device with an organic layer [36]. Optoelectronically, in the reflection configuration, both the emitter and the receiver must be located on the same side of the device. From a geometrical point of view, this is achieved by positioning the emitter–detector plane around the device’s antiprincipal planes (as defined in Section 2.3). Additionally, the PS can still work in transmission. This transmissive channel can be used as a reference that is not affected by local variations in reflectance on the functionalized side. This is why we have also considered the placement of a reference detector behind the PS. As the diffractive lens operates in reflection mode, all design variables were optimized under this configuration. The results obtained from the geometrical model in Section 2.3 have been refined to take into account diffraction and the laser beam propagation characteristics [41]. The light delivered by the emitter is modeled as a Gaussian beam displaced transversely at a distance x=−4 mm, and having a Gaussian width $\omega_{0}=10$ μm, located on the emitter plane. The transversal displacement allows the emitter and detector to fit on the same electronic board. On the other hand, the selection of the beam waist is made to properly fill the aperture of the PS. A much smaller beam waist would spill the irradiance out of the diffractive element, and a much larger one would use a reduced portion of the PS, diminishing the retrieved signal. The beam reaches the PS and the spatially modulated amplitude is propagated up to the detector plane. As the emitter and detector are on the same plane, we have varied the location of this plane to check at which distance we find the highest value of irradiance on the detector plane. This diffractive evaluation uses “Diffractio” to propagate the electric field in vacuum, but considering the wavelength in the medium, $\lambda =\lambda_{0}/n_{\mathrm{med}}$. Thus far, Diffractio operates in transmission mode; therefore, when assessing the performance of the reflective mask, we have treated the masks shown in Figure 2a,b as transmission masks.

**Figure 4:**
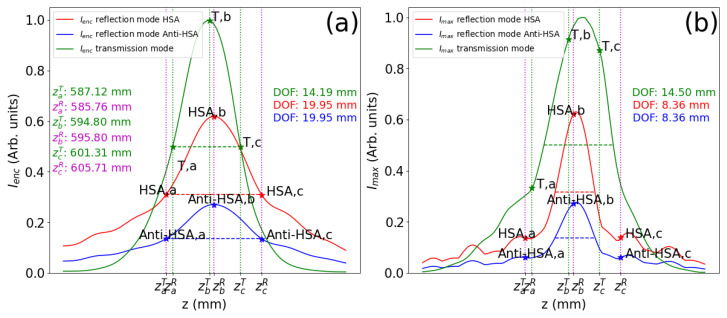
(**a**) Evolution, along the *z* axis, of the signal integrated on the detector for the three masks in Figure 2. The signals for the transmittance (T), HSA reflectance, and Anti-HSA reflectance are plotted in green, red, and blue, respectively. The locations of the maximum values are marked as $z_{b}$ for the transmission and reflection modes, which are denoted with the ^*T*^ and ^*R*^ superscripts, respectively. Additionally, we have obtained the points defining the DOF as the FWHM of the curves limited by the dotted vertical lines. The value of the DOF is given as $\mathrm{DOF}=z_{c}-z_{a}$. These points for $I_{\mathrm{enc}}$ are also plotted in (**b**) as stars, where we have represented the value of the maximum irradiance on the plane as a function of *z*. The meaning of *z* is different for the transmission and reflection modes of operation. When working in reflection, *z* describes the location of the emitter–detector plane with respect to the photon sieve. The position of the maximum obtained for $I_{\mathrm{enc}}$ is considered as the optimum location. For transmission, the emitter is located at this optimum position, and only the detector moves.

The optimum position of the emitter and detector is obtained from the evolution of the irradiance maps when moving this plane. Numerically, we have set this distance as z=595.8 mm, where the irradiance integrated on a circular aperture 100 μm in diameter reaches its maximum value. This aperture mimics the detector. We observe that the maximum occurs at a distance displaced from the position of the geometrical antiprincipal planes, $z_{{\mathrm{AH}}^{\prime }}=532$ mm. This discrepancy is mainly caused by the Gaussian beam propagation characteristics [41] and diffractive effects. The red and blue plots in Figure 4a,b represent the signals retrieved on the detector, $I_{\mathrm{enc}}$, and the maximum signal on the detection plane, $I_{\max}$, respectively. The axial propagation variable, *z*, describes the position of the emitter–detector plane for the HSA and Anti-HSA reflectances. For the transmission mode, *z* locates the detection plane, assuming that the emitter is fixed at the optimum position calculated in reflection mode. We have defined the size of the focusing region as the range in *z* where the signal is larger than half the maximum (FWHM) obtained at $z_{b}$. This region is parameterized with the depth of focus length, which is given as $\mathrm{DOF}=z_{c}-z_{a}$ in these plots. As expected, the curves for the HSA (in red) and Anti-HSA (in blue) reflectances are proportional, and have the same DOF (and the same values of $z_{a}$, $z_{b}$, and $z_{c}$). This figure also shows, as a green line, the $I_{\mathrm{enc}}$ and $I_{\max}$ around the focusing region when the PS works in transmission (denoted as T). In this case, the emitter is located at the optimum distance obtained from the reflection mode, $z_{b}=595.8$ mm, and we allow the detector to move. To better understand the ratio between signals, we have normalized the plots to the maximum value, which happens for the transmissive mode. The evolution of the maximum irradiance, $I_{\max}$, slightly departs from $I_{\mathrm{enc}}$ due to the spatial variations observed around the maximum. In Figure 4b, we have also marked with stars the locations of the points defining the DOF that was obtained from the $I_{\mathrm{enc}}$ plots.

**Figure 5:**
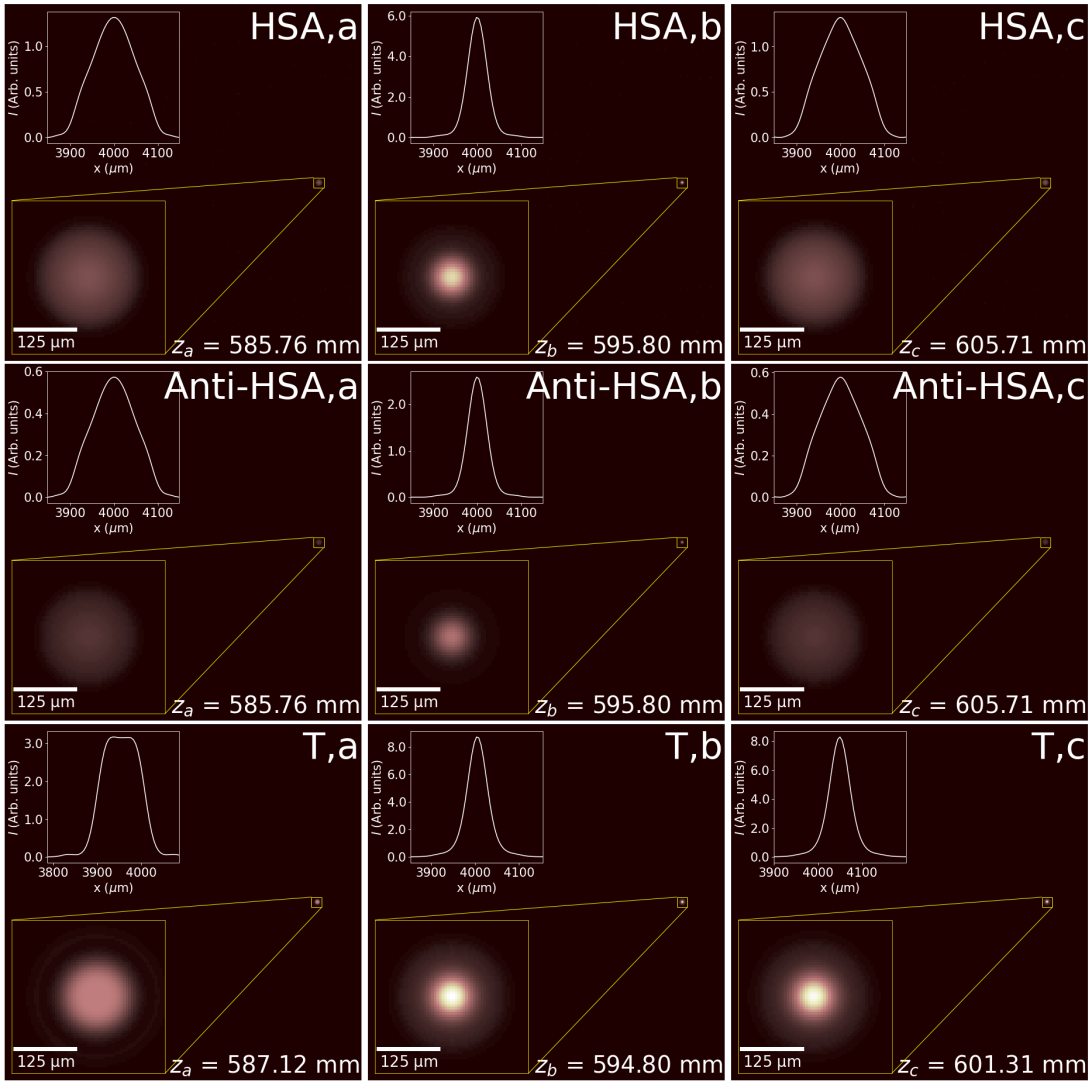
Irradiance distributions for the PS mask working in the three situations included in Figure 2 organized as rows. The columns correspond to three axial locations—$z_{a}$, $z_{b}$, and $z_{c}$—defining the focused region. The inset at the top left shows a profile of the maximum, and the inset at the bottom left zooms into the maximum of the irradiance distributions.

The values obtained from the irradiance maps are used to understand how the signals vary with the presence of the biomarker. In this case, the effect is a change in reflectance, from $\rho_{\mathrm{HSA}}=0.705$ to $\rho_{\mathrm{Anti}-\mathrm{HSA}}=0.308$. This variation is the main factor responsible for the variation in the signal. Table 1 shows the values, normalized to the maximum signal obtained in transmission, along with the axial location of these maximum values and the associated DOF, for both the $I_{\mathrm{enc}}$ and $I_{\max}$ curves. The points $z_{a},z_{b}$, and $z_{c}$ correspond to the minimum, central, and maximum *z* values defining the DOF for the $I_{\mathrm{enc}}$ curve, respectively. They are also represented in Figure 4. Figure 4 shows that, for the reflectance mode, $I_{\mathrm{enc}}$ varies more smoothly than $I_{\max}$. This means a shorter DOF when considering the maximum value of irradiance, as presented in Table 1. The irradiance maps represented in Figure 5 show how light is confined around the location of the image of the detector, calculated using diffraction. The insets of each subplot show the irradiance profile and the zoomed-in irradiance distributions. Also, we have denoted the plane *z* where these maps are evaluated. We can confirm that, although the irradiance distribution varies significantly for the selected planes, the presence of the maximum irradiance is quite noticeable when moving along the propagation axis. The values and dependences shown in Figure 4 and Table 1 have been obtained numerically. A possible uncertainty analysis of the calculated performance can be conducted by analyzing many realizations of the mask, including variations in the geometrical parameters. However, we believe that the optical bench calibration of a real device, which incorporates unavoidable uncertainties and deviation from its nominal parameters, better describes the expected changes in the signal’s level and in the optimum position for the emitter and detector [35].

The objective of this system is to serve as a detector of biomarkers for running fluids. The change in reflectance when the functionalized surface interacts with the biomarker of interest (in this case, the HSA protein) is transduced as a signal proportional to the irradiance (integrated on a detector, or as the maximum signal when an image detector array is used). Considering the values given in Table 1, we may observe that the ratio of the signal between the HSA and Anti-HSA states is around ×2.3, meaning that the signal drops around 43% when the biomarkers are fixed to the functionalized surface. This significant reduction in signal can also be monitored using another detector that retrieves the signal behind the PS in transmission mode. As expected, this ratio almost coincides with the quotient of the reflection coefficient shown in Figure 1a, that is, 0.44. The slight discrepancy comes from the departure from uniformity caused by Gaussian beam distribution that fills the aperture of the PS.

## 4. Conclusions

The analysis of flowing fluids requires the use of permeable components that permit the passage of gases or liquids. In this work, we have proposed a design for such a component and evaluated its performance in the detection of human serum albumin. To achieve this, we combined two key approaches: the functionalization of an aluminum surface to immobilize the target protein while preserving acceptable optical reflectance, and the specialized design of photon sieves that operate as permeable diffractive optical elements.

The primary optical parameter considered following the functionalization of the aluminum surface is the change in reflectance associated with immobilizing the target biomarker. In our case, this change occurs in the red region of the visible spectrum, at $\lambda_{0}=763$ nm, where the ratio between the reflectances of the HSA and Anti-HSA states is 0.44. The implementation of a functional device, however, must account for the wavelength shift introduced when light propagates through a medium with refractive index $n_{\mathrm{med}}$. Assuming $n_{\mathrm{med}}=1.35$, a value close to that of water, the design wavelength becomes $\lambda =\lambda_{0}/n_{\mathrm{med}}=565$ nm.

With the design wavelength established, an optimized photon sieve can be generated using diffraction principles and dedicated numerical analysis. We employed a Ring-by-Ring optimization algorithm that iteratively adds concentric rings of holes until the aperture is fully populated. In this study, the photon sieve was optimized for reflective operation while also allowing for transmitted light to serve as a potential reference signal.

Additional considerations include the interface between the fluid-filled tube and the photon sieve, as well as the potential use of an auxiliary lens to reduce the overall system length. A complete analysis of the device requires detailed modeling of the imaging system within the framework of geometrical optics. A practical solution is to place the emitter and detector at the antiprincipal planes, which provide a lateral magnification of $\beta^{\prime }=-1$, and thereby yield a compact and robust configuration. Incorporating diffraction and Gaussian beam propagation introduces further refinements, and numerical simulations were used to adjust the emitter–detector plane to maximize the detected signal.

Once the photon sieve has been optimized and the optical system appropriately configured, it becomes necessary to evaluate the performance of the diffractive optical element. This assessment yields the signal levels that can be recovered by a detector integrating the irradiance over a specified area. The results indicate a significant decrease in irradiance of approximately 0.43, which—as expected—closely matches the reflectance ratio.

In summary, we conclude that photon sieves—when properly optimized to maximize the signal at the detection plane—and the tailored functionalization of their reflective surfaces are essential components for monitoring flowing fluids. The design presented here can be readily adapted to a wide range of fluidic environments, and the functionalization strategy demonstrated for HSA can be extended to other biomarkers and substrates. Collectively, these developments pave the way for the future implementation of compact and robust optical sensing platforms.

## Figures and Tables

**Table 1:** Maximum integrated irradiance and maximum irradiance ($I_{\mathrm{enc}}$ and $I_{\max}$) normalized to the maximum value obtained in transmission (T), and depth of focus (DOF).

	$z_{b}$ [mm]	HSA	Anti-HSA	T
$I_{\mathrm{enc},\mathrm{ref}}$	595.80	0.62	0.27	
$I_{\mathrm{enc},\mathrm{tran}}$	594.80			1.00
$I_{\max,\mathrm{ref}}$	596.30	0.63	0.28	
$I_{\max,\mathrm{tran}}$	597.80			1.00
$z_{a}$ [mm]		585.76	585.76	587.12
$z_{c}$ [mm]		605.71	605.71	601.31
DOF [mm] ($I_{\mathrm{enc}}$)		19.95	19.95	14.19
DOF [mm] ($I_{\max}$)		8.36	8.36	14.50

## Data Availability

Data corresponding to the experimental values of reflectance of the functionalized media represented in Figure 1, and the variables and maps plotted in Figure 2, Figure 4 and Figure 5 and presented in Table 1 are fully available upon request to the authors.
